# How Do Reference Points Influence the Representation of the N200 for Consumer Preference?

**DOI:** 10.3389/fpsyg.2021.645775

**Published:** 2021-06-24

**Authors:** Guangrong Wang, Jianbiao Li, Chengkang Zhu, Shenru Wang, Shenzhou Jiang

**Affiliations:** ^1^Neural Decision Science Laboratory, School of Economics and Management, Weifang University, Weifang, China; ^2^Institute for Study of Brain-Like Economics, School of Economics, Shandong University, Jinan, China; ^3^Department of Economics and Management, Nankai University Binhai College, Tianjin, China; ^4^School of Mechanical Engineering and Automation, Beihang University, Beijing, China; ^5^School of Business Administration, Guangxi University of Finance and Economics, Nanning, China

**Keywords:** preference, N200, reference point, neuromarketing, event-related brain potential

## Abstract

Recent studies have suggested that event-related brain potential (ERP) can represent consumer preference, and there is consensus that the N200 is the best indicator of consumer preference. Measurement of reference-dependent consumer preference, in turn, requires a reference point, but it remains largely unknown how reference points modulate the preference-related N200. We designed an experiment to investigate how reference points affect the N200 based on classical paradigms. In the single-reference condition, one product was displayed in each trial; in the conjoined-reference condition, a pair of products was displayed simultaneously. Our results showed that in the single-reference condition, low-preference products elicited more negative N200 than high-preference products, replicating previous results, but the N200 could not distinguish between low‐ and high-preference products when viewing two options of similar subjective value in the conjoined-reference condition. These findings suggest that reference points modulate the representation of the N200 on consumer preference. When only viewing one product, participants make a value judgment based on their expectations. However, when viewing two products simultaneously, both their expectation and the alternative product can serve as reference points, and whether the N200 can represent consumer preference depends on which reference point is dominant. In future research, reference points must be controlled when the N200 is used to explore value-related decision-making.

## Introduction

Evaluating consumer preference for products is an important issue in marketing because preference is one of the critical factors affecting consumer decision-making. Both conscious and subconscious opinions affect consumers’ decision-making, but subconscious motivations play a critical role (Agarwal and Dutta, 2015). Traditional preference elicitation methods, such as surveys, interviews, and questionnaires, obtain consumer preference through open and conscious reports. These traditional methods have been demonstrated to generate biased or inaccurate results and not assess subconscious opinions affecting consumer behavior (Johansson et al., 2006; Blumenschein et al., 2010; Agarwal and Dutta, 2015; Telpaz et al., 2015).

Neuroscience methods can elicit subconscious aspects of consumer preferences that are not apparent in traditional tools (e.g., questionnaires; Telpaz et al., 2015; Aldayel et al., 2020). The event-related brain potential (ERP) is a common neuroscience technique with several advantages, including high temporal resolution and much lower cost than fMRI, and is non-invasive and harmless. Many ERP studies have suggested relatively robust links between ERP components and their underlying cognitive activities, which has led to identifying the physiological factors influencing behavior and preference (Levy and Glimcher, 2012; Camerer and Yoon, 2015). Therefore, a handful of studies have investigated whether ERP components index consumer preference for products.

Although limited, existing studies have suggested that several ERP components can index preferences for consumer products (Junghöfer et al., 2010; Pozharliev et al., 2015; Telpaz et al., 2015; Schaefer et al., 2016). Telpaz et al. (2015) showed that the N200 was sensitive to consumer preference, with more negative amplitude for low‐ than for high-preference products. However, they did not find the sensitivity of late potentials (e.g., P300) to consumer preference. Goto et al. (2017) found that both the N200 and late positive potential (LPP) could index consumer preference, with a more negative N200 and less positive LPP for less-preferred than for highly-preferred products. Goto et al. (2019) further explored whether ERP components measured in response to a single consumer item could predict preference for this item. Their results showed that N200, LPP, and positive slow waves (PSW) could predict consumer preference for a product with an overall accuracy of 71%, although prediction accuracy varied for different components. Ma et al. (2018) found that the N200 and LPP were sensitive to the preferences of the consumers, and Tyson-Carr et al. (2018) revealed that low-value items elicited a more pronounced N200 than high-value items.

Event-related brain potential studies have consistently suggested that the N200 could index consumer preference, with more negative amplitude for low‐ compared with high-preference products. However, results have been inconsistent for later ERP components. These findings suggest that the N200 is the best indicator of consumer preference.

These preference elicitation paradigms for ERP measurement can be summarized into two categories. First, one product is viewed in each trial, and participants are asked to make responses according to their judgment (Telpaz et al., 2015; Goto et al., 2017; Ma et al., 2018; Tyson-Carr et al., 2018). Second, two products are displayed simultaneously in each trial, and participants are required to choose which product they prefer (Li et al., 2012; Larsen and O’Doherty, 2014; Gui et al., 2016; Goto et al., 2019).

Although the ERP components can be used as a “common scale” to compare the value of products, evaluating the value requires reference points. In the first paradigm described above, in which only one product is presented in each trial, the expectation of a participant, which is formed by history and experience, can serve as the reference point. In the second paradigm, two products are displayed simultaneously, and ERP components can measure the value of a given option. Therefore, both the expectation and the alternative can influence the valuation process.

Outcomes are commonly perceived as positive or negative concerning a reference point, and expectation can sever as a reference point (Tversky and Kahneman, 1981; Yu et al., 2007). Existing studies have suggested that the N200 evoked by identical events can differ depending on the reference point (Holroyd et al., 2004a; Yu et al., 2007). For instance, the outcomes with no reward elicited an N200 when the alternative outcomes were rewards, whereas the identical outcomes did not elicit an N200 when the alternative outcomes were monetary losses (Holroyd et al., 2004a). Therefore, the N200 is generated by unfavorable feedback, but the reference point determines what constitutes unfavorable feedback.

Although studies using these paradigms have consistently suggested that the N200 can index preference, it remains largely unknown how reference points modulate the N200. In the second paradigm, both the expectation and the alternative can serve as the reference point. Since different reference point means different N200, which reference point dominates the N200 in the second paradigm? Which paradigm can better elicit consumer preference, for two categories of paradigms described above?

To examine these issues, this study explored how the expectation and the alternative influence the representation of the N200 on consumer preference. In our experiment, we studied the N200 effect in two paradigms using the same products. In the single-reference condition, only one product was viewed in each trial; in the conjoined-reference condition, a pair of products was displayed simultaneously.

The N200 is considered representative of early evaluation based on a binary classification of good vs. bad events among the salient dimension, and other features of the stimulus can modulate the N200 effect (Hajcak et al., 2006; Wu et al., 2012). If a feature enhances the salience of this dimension, the N200 effect becomes larger; if a feature diminishes the salience of this dimension, the N200 effect is reduced (Liu et al., 2014). Therefore, we predicted that the N200 effect for consumer preference would be diminished when viewing two products.

## Materials and Methods

### Participants

A total of 17 right-handed undergraduates [10 females and 7 males; ages 19–24 years, mean 21.05 years (*SD* = 1.36)] were recruited. All participants had normal or corrected-to-normal visual acuity and no history of neurological or mental disease. All participants signed informed consent forms before the experiment, which was performed per the Declaration of Helsinki and was approved by the Ethics Committee of the School of Economics, Shandong University, China. The participants received an average of 60 Chinese yuan (approximately $9.23) in compensation.

### Task and Stimuli

Our experiment consisted of three phases: (1) product rating, (2) single reference, and (3) conjoined reference. An electroencephalogram (EEG) was recorded while the participants completed phases (2) and (3).

The participants were required to give value ratings to 45 kinds of fruits in the product rating phase. First, the participants were briefly shown all items to familiarize them with the stimuli. Afterward, they indicated how much they preferred each fruit using a Likert scale with a rating of −3 (*most disliked*) to 3 (*most liked*) with unitary increments. The stimuli were then divided into two groups based on the participant’s score. The high-preference group consisted of the most-preferred five types of fruits, while the low-preference group consisted of the least-preferred five types of fruits.

In the single reference phase, in each trial, the participants were shown a picture of one fruit selected at random from the two groups of fruits (high‐ and low-preference groups). Each type of fruit was repeated 10 times, yielding 100 trials. The participants were instructed to report their rating for the fruit shown as either high or low.

In the conjoined reference phase, two fruit pictures were displayed simultaneously on the screen. Each fruit was paired with fruit from the same group without duplication, resulting in 10 pairs for each group. Each pair was repeated five times, yielding 100 trials. The participants were instructed to indicate which of the two options they preferred.

At the beginning of the experiment, the participants were told that the market value of the fruit in each trial was nearly identical. The subsequent post-experimental questionnaire revealed that all participants believed this statement.

### Procedure

The participants were instructed about the rules of the experiment task through an explanation of the written instructions. The task was performed in a quiet and isolated laboratory. The participants were told that they would be paid for their participation after completing the experiment. The recording session took approximately 30 min.

**Figure 1:**
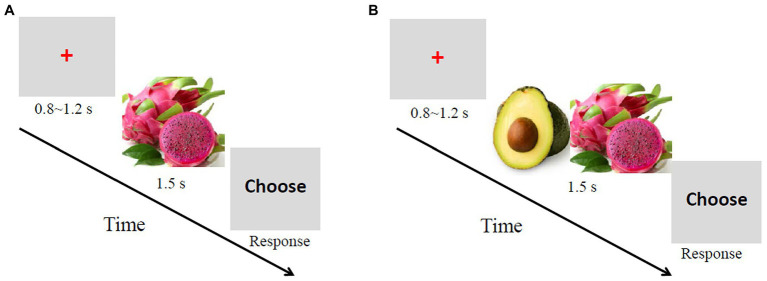
Sequence of trial events. **(A)** Single-reference condition. The task was to judge whether the fruit presented was of high or low value. **(B)** Conjoined-reference condition. The task was to judge which fruit was of higher value.

Each trial in the single-reference condition adopted the following sequence. A red cross was first presented in the center of the screen for 800–1,200 ms. Then, a picture of a randomly selected fruit was displayed for 1,500 ms. Next, the word “choose” appeared until the participants made a choice. The participants were required to indicate whether the fruit was of high or low subjective value by pressing either the right key of the mouse to denote a high value or the left key to denote a low value. After a blank screen was displayed for 1,000 ms, the next trial began (Figure 1). A total of 100 trials were randomly divided into two blocks of 50 trials each.

The procedure in the conjoined-reference condition was the same as the single reference condition except that two fruit pictures were displayed simultaneously. The orders of pictures were randomly assigned, and the positions of pictures were randomly assigned (left or right) on each trial and counterbalanced across trials. The participants had to indicate which fruit was of higher subjective value by pressing either the left key of the mouse to denote that the value of the fruit1 (i.e., the fruit shown on the left) was higher or the right key to denote that the value of the fruit2 was higher.

### EEG Recording and Analysis

Electroencephalograms were acquired continuously at a 1,000 Hz sampling rate with a Neuroscan Synamp2 Amplifier using an electrode cap with Ag/AgCl electrodes mounted according to the extended international 10–20 system. The EEG signals were amplified online (bandpass: 0.05–0.100 Hz). All electrode recordings were referenced online to the left mastoid and re-referenced offline to the left and right mastoid average. Electrode impedance was kept under 5 kΩ. Following electrode application, the participants sat in a comfortable chair in a shielded room and were asked to fixate on the center of a computer display located 1 m away from their eyes.

Electroencephalogram epochs of 1,000 ms (from −200 to 800 ms after the onset of stimulus) were extracted offline, and the 200 ms prestimulus was defined as a baseline. Data were then corrected for ocular artifacts with an algorithm implemented in the Neuroscan software (Curry 7, Compumedics, El Paso, TX, United States).^1^ Trials contaminated by amplifier clipping, bursts of electromyographic activity, or peak-to-peak deflection exceeding ±75 μV were excluded from further analysis. The remaining trials were baseline corrected. The EEG segments were averaged separately for product type (high vs. low preference). The averaged ERPs were digitally filtered with a low-pass filter at 30 Hz. A within-subjects repeated measure of ANOVA was used to analyze the ERP data.

According to classical definitions, the N200 is an ERP component occurring in the time window of 250–350 ms after the onset of the stimulus. Our visual inspection of waveforms showed that the N200 had its maximum at Fz at approximately 330 ms. Therefore, the mean amplitudes between 300 and 350 ms after offer onset were analyzed for the N200. Consistent with previous research, the peak potential of the N200 was distributed on the prefrontal scalp areas; therefore, we selected six electrodes from the prefrontal scalp (F3, Fz, F4, FC3, FCz, and FC4) for statistical analysis.

Behavioral and ERP data were analyzed using SPSS (version 22, SPSS Inc., Chicago, IL, United States). A Greenhouse–Geisser correction for the violation of sphericity assumption was applied when the degrees of freedom exceeded one. The significance level was set at *p* < 0.05 for all analyses.

## Results

### Behavioral Results

In the single-reference condition, participants’ decisions were per their ratings of the fruits. Participants tended to assign high (low) values for the presented fruits after assigning high (low) ratings in the previous phase.

In the conjoined-reference condition, the participant’s valuation of two options was almost the same. The average choices for all participants on the left presented fruit, and right one were 49 and 51%, respectively, for the high-preference group and were 48 and 52%, respectively, for the low-preference group. These results were also consistent with the predictions of rating results.

Mean response times ± standard errors of choices were 492 ± 19 ms and 627 ± 34 ms, respectively, for single‐ and conjoined-reference conditions. Mean response times in the conjoined-reference condition were significantly larger than in the single-reference condition (*T* = −3.336, *p* = 0.004).

### ERP Results

#### Single-Reference Condition

**Figure 2:**
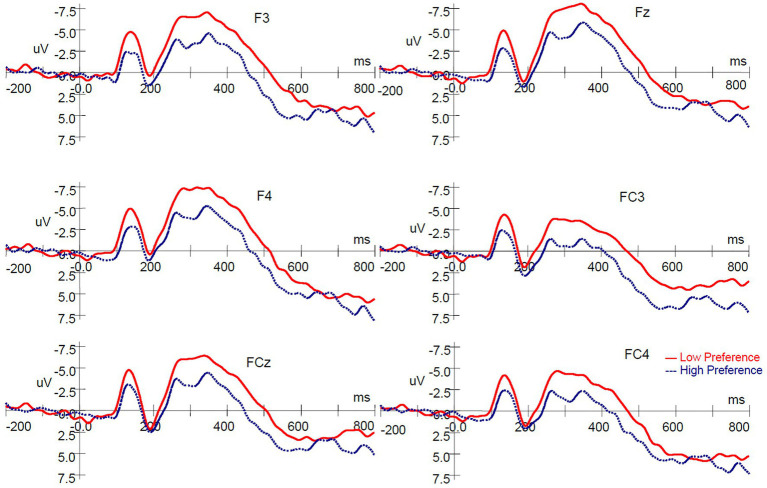
ERP responses time-locked to the onset of stimuli in the single-reference condition. ERP, event-related brain potential.

**Figure 3:**
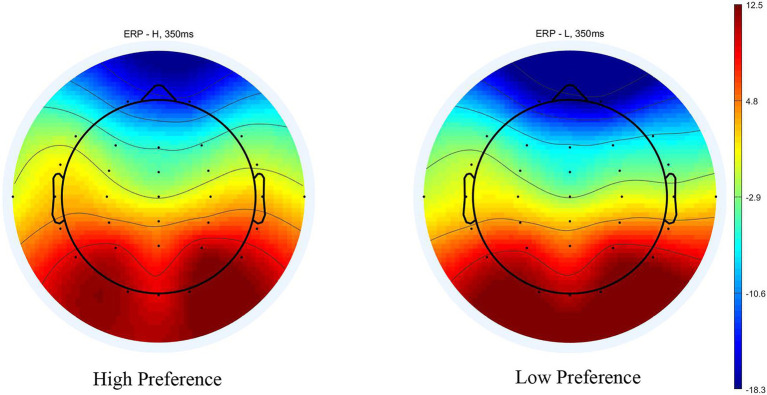
Scalp topography (350 ms) in the single-reference condition.

To define neural responses to product type, a 2 (product type: high‐ vs. low-preference) × 6 (electrodes: F3, Fz, F4, FC3, FCz, and FC4) repeated measures ANOVA was conducted on the mean amplitude of the N200. The ERP waves evoked by high‐ and low-preference products are shown in Figure 2. The results revealed significant main effects of product type [*F*(1, 16) = 25.749, *p* < 0.001, *η_p_*^2^ = 0.617] and electrodes [*F*(5, 80) = 13.772, *p* < 0.001, *η_p_*^2^ = 0.463]. A significant product type × electrodes interaction was not observed [*F*(5, 80) = 0.993, *p* = 0.403, *η_p_*^2^ = 0.058]. Low-preference products elicited a more negative-going deflection than did high-preference products (Figures 2, 3).

The scalp voltage maps showed strong positive activity in the occipital areas, so a 2 (product type: high vs. low preference) × 3 (electrodes: O1, Oz, and O2) repeated measures ANOVA was conducted on the mean amplitude as same time windows as the frontal-N200. There was no significant main effect of product type [*F* (1, 16) = 0.176, *p* = 0.680, *η_p_*^2^ = 0.011], and no significant interaction between product type and electrodes [*F*(2, 32) = 0.717, *p* = 0.441, *η_p_*^2^ = 0.043], although a main effect of electrodes [*F*(2, 32) = 13.939, *p* < 0.001, *η_p_*^2^ = 0.466] was observed.

#### Conjoined-Reference Condition

**Figure 4:**
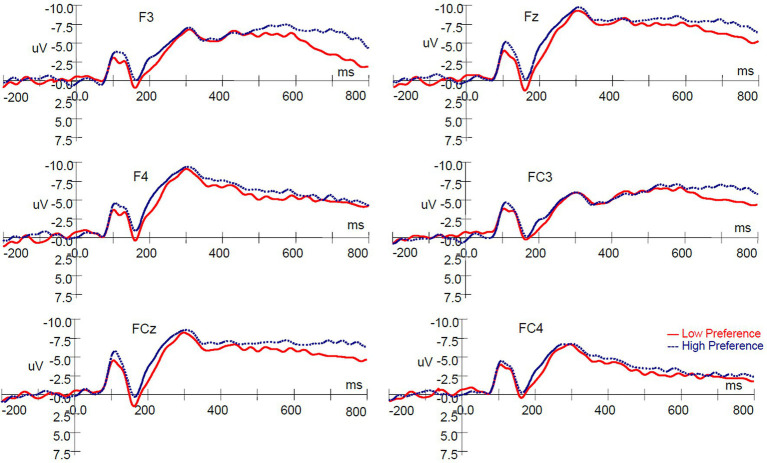
ERP responses time-locked to the onset of stimuli in the conjoined-reference condition.

**Figure 5:**
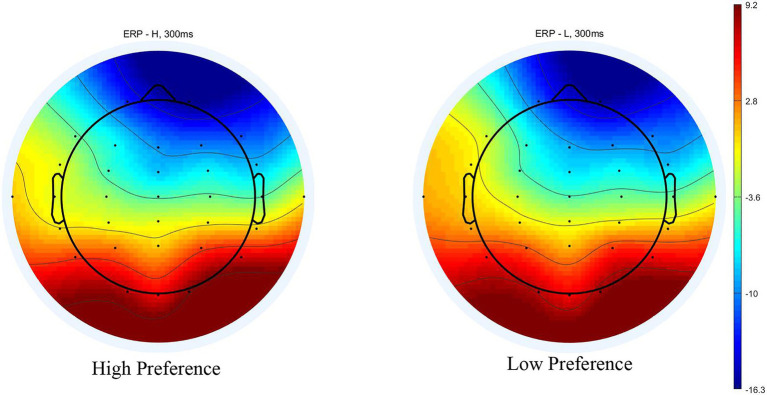
Scalp topography for high‐ and low-preference at 300 ms in the conjoined-reference condition.

The ERPs evoked by paired products are shown in Figure 4. A 2 (product type: high‐ vs. low-preference) × 6 (electrodes: F3, Fz, F4, FC3, FCz, and FC4) repeated measures ANOVA was conducted on the mean amplitude of the N200. There was no significant main effect of product type [*F*(1, 16) = 0.425, *p* = 0.524, *η_p_*^2^ = 0.026], and no significant interaction between product type and electrodes [*F*(5, 80) = 0.314, *p* = 0.802, *η_p_*^2^ = 0.019]. A significant main effect of electrodes [*F*(5, 80) = 5.138, *p* = 0.022, *η_p_*^2^ = 0.243] was found (Figures 4, 5).

The scalp voltage maps showed strong positive activity in the occipital areas, so a 2 (product type: high vs. low preference) × 3 (electrodes: O1, Oz, and O2) repeated measures ANOVA was conducted on the mean amplitude as same time windows as the frontal-N200. There was no significant main effect of product type [*F* (1, 16) = 0.033, *p* = 0.859, *η_p_*^2^ = 0.02], and no significant interaction between product type and electrodes [*F*(2, 32) = 0.510, *p* = 0.5241, *η_p_*^2^ = 0.031]. A significant main effect of electrodes [*F*(2, 32) = 6.098, *p* < 0.013, *η_p_*^2^ = 0.276] was found.

### Time-Frequency Analysis

#### Single-Reference Condition

In addition to the ERP component, event-related spectral perturbation (ERSP) analysis was conducted to explore the relationship between a specific frequency domain within the EEG signal and the subjective value of the stimulus.

**Figure 6:**
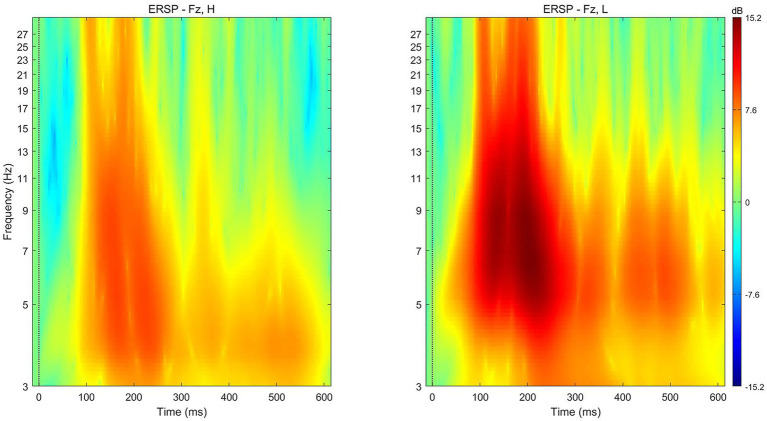
ERSP for high‐ (left) and low‐ (right) preference products at electrode Fz in the single-reference condition. ERSP, event-related spectral perturbation.

The average theta power following the presentation of low-preference products (*M* = 11.7471 dB, *SD* = 8.0224) was significantly stronger than that following the presentation of high-preference products (*M* = 6.093 dB, *SD* = 6.70981) at electrode Fz [*t* (17) = −3.302, *p* = 0.004] for the time 200–350 ms after presentation (Figure 6).

#### Conjoined-Reference Condition

**Figure 7:**
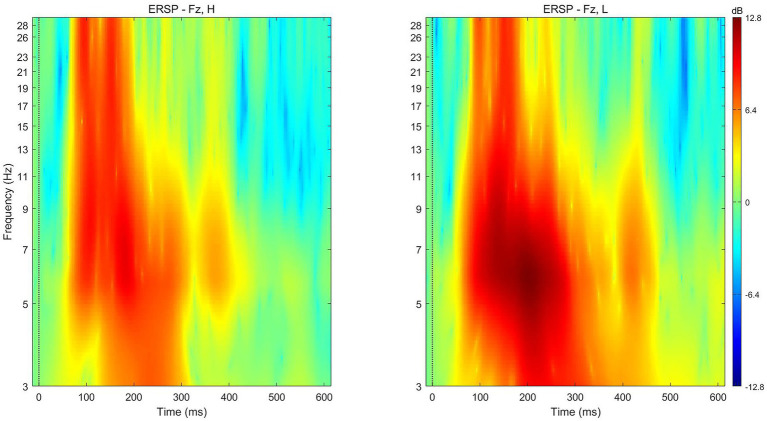
ERSP for high‐ (left) and low‐ (right) preference products at electrode Fz in the conjoined-reference condition.

The average theta power following the presentation of paired products with low subjective values (*M* = 8.8628 dB, *SD* = 5.57763) was similar to that following the presentation of paired products with high subjective values (*M* = 6.1268 dB, *SD* = 9.63887) at electrode Fz [*t* (17) = −1.055, *p* = 0.307] for the time 200–350 ms after presentation (Figure 7).

## Discussion

In this study, we explored how reference points influence the preference-related N200. When only one product was displayed in each trial, low-preference products elicited more pronounced N200 than high-preference products. When a pair of products with similar preferences was viewed simultaneously, the N200 difference between low‐ and high-preference products disappeared. This suggests that reference points can modulate consumer preference elicitation.

The anterior cingulate cortex (ACC) can represent participants’ expectation of rewards with particular stimulus features, even though these features are unrelated to actual rewards (Yeung et al., 2005; Telpaz et al., 2015; Schaefer et al., 2016). Events against expectation can trigger the modulation of dopaminergic activity in the ACC, which is then reflected in the N200. Previous studies have demonstrated that unfavorable events elicit a more negative N200 than favorable events (Hajcak et al., 2006; Simons, 2010; Kreussel et al., 2012). It is human nature to seek advantages and avoid disadvantages, and by definition, people appreciate high-value items. Therefore, when viewing a single object for value judgment, an individual may expect it to be a high-value item (i.e., a good item), and this expectation may be used as a reference point in assigning value to that object. That low-preference product elicited more pronounced N200 than high-preference products in the single-reference condition is in accordance with these previous findings.

The paradigm in the single-reference condition is similar to most previous consumer preference studies (Telpaz et al., 2015; Goto et al., 2017; Ma et al., 2018; Tyson-Carr et al., 2018). In these study paradigms, only one product is presented in each trial, and participants make a value judgment using only their expectations. Studies using this paradigm have consistently shown that the N200 following presentation of low-preference products was more negative than the N200 following high-preference products. Our results in the single-reference condition replicated previous findings.

Gehring et al. (2012) extracted theta oscillations using the Morlet wavelet transform and found frontally focused theta (4–7 Hz) activity for monetary losses compared with monetary gains when the medial frontal negativity (MFN) was evoked. Billeke et al. (2013) found that theta activity, which reflects the activity of dACC, was modulated by the individual strategy of the study participant. Telpaz et al. (2015) found a correlation between the theta band power and participants’ rank-ordered preferences without requiring any hemispheric asymmetry. They demonstrated that predictive power depends on the magnitude of the theta power band, suggesting a cardinal scale for their measurement. Moreover, they demonstrated that the signal likely originates from frontal areas.

The N200 and feedback-related negativity (FRN) are negative deflections at fronto-central recording sites, and they peak between 250 and 350 ms post-presentation of stimuli (Nieuwenhuis et al., 2004a; Holroyd and Krigolson, 2007). The N200 and the FRN share similar scalp distributions, time courses, morphologies, and functional dependencies (Miltner et al., 1997; Gehring and Willoughby, 2002; Yeung et al., 2004; Telpaz et al., 2015). Source localization studies have demonstrated that both the N200 and the FRN are generated in ACC (Miltner et al., 1997; Gehring and Willoughby, 2002; Yeung et al., 2004), and this notion has been supported by fMRI research (Holroyd et al., 2004b; Huettel and McCarthy, 2004; Mars et al., 2005) and intracranial studies (Halgren et al., 2002; Wang et al., 2005). Therefore, scholars have suggested that they may be the same phenomenon (Toyomaki and Murohashi, 2005; Holroyd et al., 2006, 2008; Holroyd and Krigolson, 2007; Kamarajan et al., 2009; Walsh and Anderson, 2012; Xiong et al., 2014; Telpaz et al., 2015). Holroyd et al. (2006) verified this notion using a guessing task and an oddball task, and Holroyd et al. (2008) replicated their 2006 finding using an oddball task and a time estimation task. Therefore, the findings on the FRN can be used to explain the N200.

The FRN is considered to represent an evaluation of stimuli along a general good-bad dimension, with more negative amplitude following unfavorable compared with favorable outcomes (Hajcak et al., 2006; Kreussel et al., 2012). However, several studies questioned this and suggested that the FRN is sensitive to unlikely outcomes, regardless of their valence (Oliveira et al., 2007; Ferdinand et al., 2012). These studies have also suggested that for a stimulus conveying multiple dimensions of information, the FRN is sensitive to the most perceptually salient information and insensitive to the same information when it is not perceptually salient (Nieuwenhuis et al., 2004b; Liu and Gehring, 2009; Liu et al., 2014).

In our conjoined-reference condition, when two products were displayed simultaneously, participants had to judge which product was more valuable based on two reference points: their expectation and the alternative product. Because the two options were of similar subjective value, participants had to pay more attention to distinguish them. Therefore, the value of the alternative became a salient reference point, and the expectation was no longer a salient reference point. So, the N200 did not reflect the difference between low‐ and high-preference products in the conjoined-reference condition.

Contrary to our findings, several studies with a similar study paradigm to our conjoined-reference condition found that the N200 could represent preference (Li et al., 2012; Gui et al., 2016; Goto et al., 2019). This inconsistency may be the result of participants using different reference points. In previous study paradigms, low-preference products were paired with high-preference products. The value difference between the two options was large enough to make it easy to distinguish between them in these trials. The low decision conflict forced participants to use the expectation as the salient reference point. Therefore, the N200 can index consumer preference. In the conjoined-reference condition, both the alternative option and the expectation serve as reference points. If the expectation serves as a main reference point, a less-than-expected product will elicit more negative N200. Therefore, N200 can represent consumer preference. However, the small difference between the two options would result in high decision conflict, in which the alternative option would serve as the main reference point. Thus, N200 cannot represent consumer preference in these scenarios. In summary, both of the paradigms in the present study can be used to distinguish between high and low consumer preferences if appropriate reference points are selected.

Previous studies have implicated the ACC in conflict monitoring during perceptual tasks (Botvinick et al., 2001; Greene et al., 2004; Stauffer et al., 2014). An fMRI study by Pochon et al. (2008) revealed that the ACC indeed indexed conflict at the decision stage. They found that facing difficult (high conflict) decisions led to increased ACC activity relative to easier (low conflict) decisions. In our experiment, because all paired products were of similar value, it was more difficult for participants to distinguish which product they preferred. The ACC activity reflected this high conflict, and a similar N200 was evoked irrespective of the value.

Feedback-related negativity represents early evaluation based on a binary classification of good vs. bad events among the salient dimension, and other features conveyed by the stimulus can modulate this FRN effect (Nieuwenhuis et al., 2004b; Hajcak et al., 2006; Liu and Gehring, 2009; Wu et al., 2012; Liu et al., 2014). If a feature enhances the salience of the dimension, the FRN effect becomes larger; if a feature diminishes the salience of the dimension, the FRN effect is reduced. For example, Liu et al. (2014) investigated how the perceptual properties of the feedback modulate the FRN. In their study, when the perceptual properties between gain and loss feedback were different, the FRN amplitude for loss feedback was larger relative to when perceptual properties between gain and loss feedback were similar. They suggested that incongruent perceptual properties enhanced the salience of valence and elicited a larger difference wave in the FRN. These findings support our results.

In our conjoined-reference condition, the negativity trend remained strong for the entire measurement period. This may be because when evaluating an option, in addition to effects of subjective value, decision-making is influenced by decision conflict (Pochon et al., 2008; Peters and Büchel, 2011). Decisions are difficult when options are of similar value, whereas decisions are easier when option values are different (Peters and Büchel, 2011). Studies of decision conflict monitoring have suggested that the high decision conflict condition would result in a more negative wave in the fronto-central areas 250 and 500 ms after stimulus presentation than a low decision conflict condition (Liotti et al., 2000; Van Veen and Carter, 2002; Markela-Lerenc et al., 2004; Yang and Zhang, 2011). In the conjoined-reference condition, because the two options were of similar subjective value, participants had to pay more attention to distinguish them. Therefore, decisions in the conjoined-reference condition were more difficult than those in the single-reference condition. Furthermore, in our decision task, decision-making was divided into two stages: the valuation stage and the choice stage. Mean response times in the conjoined-reference condition were significantly larger than that in the single-reference condition. This indicated that the decisions in the conjoined-reference condition were difficult and that trade-offs were made throughout the evaluation stage to affect the choice responses in subsequent stages. Therefore, the negativity trend remained strong for the entire measurement period during the valuation stage.

## Conclusion

In summary, the N200 can index consumer preference, but specific reference points influence it. When only viewing one product, participants make a value judgment based on their expectations, and the N200 is a good indicator of consumer preference. When viewing two products simultaneously, both the expectations of the participants and the alternative can serve as reference points, and whether the N200 can represent consumer preference depends on which reference point is dominant. If the value of the two options is similar, the alternative serves as the dominant reference point, and the N200 cannot reflect consumer preference. If the value of the two options is different enough to make the expectation a dominant reference point, the N200 can represent consumer preference. These findings contribute to an understanding of the neural processes underlying consumer preference. In future research, the reference points must be controlled when the N200 is used to explore value-related decision-making.

## Data Availability Statement

The raw data supporting the conclusions of this article will be made available by the authors, without undue reservation.

## Ethics Statement

The studies involving human participants were reviewed and approved by the Ethics Committee of School of Economics, Shandong University, China. The patients/participants provided their written informed consent to participate in this study.

## Author Contributions

GW and JL designed this study and wrote the manuscript. CZ and SJ implemented the experimental protocols and collected the data. SW and GW analyzed the data. All authors contributed to the article and approved the submitted version.

### Conflict of Interest

The authors declare that the research was conducted in the absence of any commercial or financial relationships that could be construed as a potential conflict of interest.

## Footnotes

^1^We also removed ocular artifacts using independent component analysis (ICA) as advised by Jung et al. (2000). There was no significant difference in ERP results between the two methods.

## References

[ref1] Agarwal S. Dutta T. (2015). Neuromarketing and consumer neuroscience: current understanding and the way forward. Decision 42, 457–462. 10.1007/s40622-015-0113-1

[ref2] Aldayel M. Ykhlef M. Al-Nafjan A. (2020). Deep learning for eeg-based preference classification in neuromarketing. Appl. Sci. 10:1525. 10.3390/app10041525

[ref3] Billeke P. Zamorano F. Cosmelli D. Aboitiz F. (2013). Oscillatory brain activity correlates with risk perception and predicts social decisions. Cereb. Cortex 23, 2872–2883. 10.1093/cercor/bhs269, PMID: 22941720

[ref4] Blumenschein K. Blomquist G. C. Johannesson M. Horn N. Freeman P. (2010). Eliciting willingness to pay without bias: evidence from a field experiment. Econ. J. 118, 114–137. 10.1111/j.1468-0297.2007.02106.x

[ref5] Botvinick M. M. Braver T. S. Carter C. S. Barch D. M. Cohen J. D. (2001). Conflict monitoring and cognitive control. Psychol. Rev. 108, 624–652. 10.1037/0033-295X.108.3.624, PMID: 11488380

[ref6] Camerer C. Yoon C. (2015). Introduction to the Journal of Marketing Research special issue on euroscience and marketing. J. Mark. Res. 52, 423–426. 10.1509/0022-2437-52.4.423

[ref7] Ferdinand N. K. Mecklinger A. Kray J. Gehring W. J. (2012). The processing of unexpected positive response outcomes in the mediofrontal cortex. J. Neurosci. 32, 12087–12092. 10.1523/JNEUROSCI.1410-12.2012, PMID: 22933792 PMC6621524

[ref8] Gehring W. J. Liu Y. Orr J. M. Carp J. (2012). “The error-related negativity (ERN/Ne),” in Oxford Handbook of Event-Related Potential Components. eds. Luck S. J. Kappenman E. (New York: Oxford University Press), 231–294.

[ref9] Gehring W. J. Willoughby A. R. (2002). The medial frontal cortex and the rapid processing of monetary gains and losses. Science 295, 2279–2282. 10.1126/science.1066893, PMID: 11910116

[ref10] Goto N. Lim X. L. Shee D. Hatano A. Khong K. W. Buratto L. G.. (2019). Can brain waves really tell if a product will be purchased? Inferring consumer preferences from single-item brain potentials. Front. Integr. Neurosci. 13:19. 10.3389/fnint.2019.00019, PMID: 31316357 PMC6611214

[ref11] Goto N. Mushtaq F. Shee D. Lim X. L. Mortazavi M. Watabe M.. (2017). Neural signals of selective attention are modulated by subjective preferences and buying decisions in a virtual shopping task. Biol. Psychol. 128, 11–20. 10.1016/j.biopsycho.2017.06.004, PMID: 28666891

[ref12] Greene J. D. Nystrom L. E. Engell A. D. Darley J. M. Cohen J. D. (2004). The neural bases of cognitive conflict and control in moral judgment. Neuron 44, 389–400. 10.1016/j.neuron.2004.09.027, PMID: 15473975

[ref13] Gui D. Li J. Li X. Luo Y. (2016). Temporal dynamics of the interaction between reward and time delay during intertemporal choice. Front. Psychol. 7:1526. 10.3389/fpsyg.2016.01526, PMID: 27785126 PMC5060948

[ref14] Hajcak G. Moser J. S. Holroyd C. B. Simons R. F. (2006). The feedback-related negativity reflects the binary evaluation of good versus bad outcomes. Biol. Psychol. 71, 148–154. 10.1016/j.biopsycho.2005.04.001, PMID: 16005561

[ref15] Halgren E. Boujon C. Clarke J. Wang C. Chauvel P. (2002). Rapid distributed fronto-parieto-occipital processing stages during working memory in humans. Cereb. Cortex 12, 710–728. 10.1093/cercor/12.7.710, PMID: 12050083

[ref16] Holroyd C. B. Hajcak G. Larsen J. T. (2006). The good, the bad and the neutral: electrophysiological responses to feedback stimuli. Brain Res. 1105, 93–101. 10.1016/j.brainres.2005.12.015, PMID: 16427615

[ref17] Holroyd C. B. Krigolson O. E. (2007). Reward prediction error signals associated with a modified time estimation task. Psychophysiology 44, 913–917. 10.1111/j.1469-8986.2007.00561.x, PMID: 17640267

[ref18] Holroyd C. B. Larsen J. T. Cohen J. D. (2004a). Context dependence of the event-related brain potential associated with reward and punishment. Psychophysiology 41, 245–253. 10.1111/j.1469-8986.2004.00152.x, PMID: 15032989

[ref19] Holroyd C. B. Nieuwenhuis S. Yeung N. Nystrom L. Mars R. Coles M. G.. (2004b). Dorsal anterior cingulate cortex shows fMRI response to internal and external error signals. Nat. Neurosci. 7, 497–498. 10.1038/nn1238, PMID: 15097995

[ref20] Holroyd C. B. Pakzad-Vaezi K. L. Krigolson O. E. (2008). The feedback correct-related positivity: sensitivity of the event-related brain potential to unexpected positive feedback. Psychophysiology 45, 688–697. 10.1111/j.1469-8986.2008.00668.x, PMID: 18513364

[ref21] Huettel S. A. McCarthy G. (2004). What is odd in the oddball task? Prefrontal cortex is activated by dynamic changes in response strategy. Neuropsychologia 42, 379–386. 10.1016/j.neuropsychologia.2003.07.009, PMID: 14670576

[ref22] Johansson P. Hall L. Sikstrom S. Tarning B. Lind A. (2006). How something can be said about telling more than we can know: on choice blindness and introspection. Conscious. Cogn. 15, 673–692. 10.1016/j.concog.2006.09.004, PMID: 17049881

[ref23] Jung T. Makeig S. Humphries C. Lee T. Mckeown M. J. Iragui V.. (2000). Removing electroencephalographic artifacts by blind source separation. Psychophysiology 37, 163–178. 10.1111/1469-8986.3720163, PMID: 10731767

[ref24] Junghöfer M. Kissler J. Schupp H. T. Putsche C. Elling L. Dobel C. (2010). A fast neural signature of motivated attention to consumer goods separates the sexes. Front. Hum. Neurosci. 4:179. 10.3389/fnhum.2010.00179, PMID: 21079751 PMC2978038

[ref25] Kamarajan C. Porjesz B. Rangaswamy M. Tang Y. Chorlian D. B. Padmanabhapillai A.. (2009). Brain signatures of monetary loss and gain: outcome-related potentials in a single outcome gambling task. Behav. Brain Res. 197, 62–76. 10.1016/j.bbr.2008.08.011, PMID: 18775749 PMC2645043

[ref26] Kreussel L. Hewig J. Kretschmer N. Hecht H. Coles M. G. Miltner W. H. (2012). The influence of the magnitude, probability, and valence of potential wins and losses on the amplitude of the feedback negativity. Psychophysiology 49, 207–219. 10.1111/j.1469-8986.2011.01291.x, PMID: 22091824

[ref27] Larsen T. O’Doherty J. P. (2014). Uncovering the spatio-temporal dynamics of value-based decision-making in the human brain: a combined fMRI-EEG study. Philos. Trans. R. Soc. Lond. Ser. B Biol. Sci. 369:20130473. 10.1098/rstb.2013.0473, PMID: 25267816 PMC4186227

[ref28] Levy D. J. Glimcher P. W. (2012). The root of all value: a neural common currency for choice. Curr. Opin. Neurobiol. 22, 1027–1038. 10.1016/j.conb.2012.06.001, PMID: 22766486 PMC4093837

[ref29] Li J. Gui D. Feng C. Wang W. Du B. Q. Gan T.. (2012). Victims’ time discounting 2.5 years after the wenchuan earthquake: an erp study. PLoS One 7:e40316. 10.1371/journal.pone.0040316, PMID: 22792277 PMC3390369

[ref30] Liotti M. Woldorff M. G. Perez R. Mayberg H. S. (2000). An ERP study of the temporal course of the Stroop color-word interference effect. Neuropsychologia 38, 701–711. 10.1016/S0028-3932(99)00106-2, PMID: 10689046

[ref31] Liu Y. Gehring W. J. (2009). Loss feedback negativity elicited by single‐ versus conjoined-feature stimuli. NeuroReport 20, 632–636. 10.1097/WNR.0b013e32832a3250, PMID: 19293730

[ref32] Liu Y. Nelson L. D. Bernat E. M. Gehring W. J. (2014). Perceptual properties of feedback stimuli influence the feedback-related negativity in the flanker gambling task. Psychophysiology 51, 782–788. 10.1111/psyp.12216, PMID: 24673119

[ref33] Ma Y. Jin J. Yu W. Zhang W. Xu Z. Ma Q. (2018). How is the neural response to the design of experience goods related to personalized preference? An implicit view. Front. Neurosci. 12:760. 10.3389/fnins.2018.00760, PMID: 30416423 PMC6214219

[ref34] Markela-Lerenc J. Ille N. Kaiser S. Fiedler P. Weisbrod M. (2004). Prefrontal-cingulate activation during executive control: which comes first? Brain Res. Cogn. Brain Res. 18, 278–287. 10.1016/j.cogbrainres.2003.10.013, PMID: 14741314

[ref35] Mars R. B. Coles M. G. H. Grol M. J. Holroyd C. B. Nieuwenhuis S. Hulstijn W.. (2005). Neural dynamics of error processing in medial frontal cortex. NeuroImage 28, 1007–1013. 10.1016/j.neuroimage.2005.06.041, PMID: 16055352

[ref36] Miltner W. Braun C. Coles M. (1997). Event-related brain potentials following incorrect feedback in a time-estimation task: evidence for a “generic” neural system for error detection. J. Cogn. Neurosci. 9, 788–798. 10.1162/jocn.1997.9.6.788, PMID: 23964600

[ref37] Nieuwenhuis S. Holroyd C. B. Mol N. Coles M. G. H. (2004a). Reinforcement–related brain potentials from medial frontal cortex: origins and functional significance. Neurosci. Biobehav. Rev. 28, 441–448. 10.1016/j.neubiorev.2004.05.003, PMID: 15289008

[ref38] Nieuwenhuis S. Yeung N. Holroyd C. B. Schurger A. Cohen J. D. (2004b). Sensitivity of electrophysiological activity from medial frontal cortex to utilitarian and performance feedback. Cereb. Cortex 14, 741–747. 10.1093/cercor/bhh034, PMID: 15054053

[ref39] Oliveira F. T. McDonald J. J. Goodman D. (2007). Performance monitoring in the anterior cingulate is not all error related: expectancy deviation and the representation of action–outcome associations. J. Cogn. Neurosci. 19, 1994–2004. 10.1162/jocn.2007.19.12.1994, PMID: 17892382

[ref40] Peters J. Büchel C. (2011). The neural mechanisms of inter-temporal decision-making: understanding variability. Trends Cogn. Sci. 15, 227–239. 10.1016/j.tics.2011.03.002, PMID: 21497544

[ref41] Pochon J. B. Riis J. Sanfey A. G. Nystrom L. E. Cohen J. D. (2008). Functional imaging of decision conflict. J. Neurosci. 28, 3468–3473. 10.1523/JNEUROSCI.4195-07.2008, PMID: 18367612 PMC6670602

[ref42] Pozharliev R. Verbeke W. J. M. I. Van Strien J. W. Bagozzi R. P. (2015). Merely being with you increases my attention to luxury products: using EEG to understand 39 consumers’ emotional experience with luxury branded products. J. Mark. Res. 52, 546–558. 10.1509/jmr.13.0560

[ref43] Schaefer A. Buratto L. G. Goto N. Brotherhood E. V. (2016). The feedback-related negativity and the P300 brain potential are sensitive to price expectation violations in a virtual shopping task. PLoS One 11:e0163150. 10.1371/journal.pone.0163150, PMID: 27658301 PMC5033321

[ref44] Simons R. F. (2010). The way of our errors: theme and variations. Psychophysiology 47, 1–14. 10.1111/j.1469-8986.2009.00929.x, PMID: 19929897

[ref45] Stauffer W. R. Lak A. Schultz W. (2014). Dopamine reward prediction error responses reflect marginal utility. Curr. Biol. 24:2491. 10.1016/j.cub.2014.08.064, PMID: 25283778 PMC4228052

[ref46] Telpaz A. Webb R. Levy D. J. (2015). Using EEG to predict consumers’ future choices. J. Mark. Res. 52, 511–529. 10.1509/jmr.13.0564

[ref47] Toyomaki A. Murohashi H. (2005). The ERPs to feedback indicating monetary loss and gain on the game of modified “rock-paper-scissors”. Int. Congr. Ser. 1278, 381–384. 10.1016/j.ics.2004.11.032

[ref48] Tversky A. Kahneman D. (1981). The framing of decisions and the psychology of choice. Science 211, 453–458. 10.1126/science.7455683, PMID: 7455683

[ref49] Tyson-Carr J. Kokmotou K. Soto V. Cook S. Fallon N. Giesbrecht T.. (2018). Neural correlates of economic value and valuation context. J. Neurophysiol. 119, 1924–1933. 10.1152/jn.00524.2017, PMID: 29442556

[ref50] Van Veen V. Carter C. S. (2002). The timing of action-monitoring processes in the anterior cingulate cortex. J. Cogn. Neurosci. 14, 593–602. 10.1162/08989290260045837, PMID: 12126500

[ref51] Walsh M. M. Anderson J. R. (2012). Learning from experience: event-related potential correlates of reward processing, neural adaptation, and behavioral choice. Neurosci. Biobehav. Rev. 36, 1870–1884. 10.1016/j.neubiorev.2012.05.008, PMID: 22683741 PMC3432149

[ref52] Wang C. Ulbert I. Schomer D. L. Marinkovic K. Halgren E. (2005). Responses of human anterior cingulate cortex microdomains to error detection, conflict monitoring, stimulus-response mapping, familiarity and orienting. J. Neurosci. 25, 604–613. 10.1523/JNEUROSCI.4151-04.2005, PMID: 15659596 PMC6725336

[ref53] Wu Y. Zhang D. Elieson B. Zhou X. (2012). Brain potentials in outcome evaluation: when social comparison takes effect. Int. J. Psychophysiol. 85, 145–152. 10.1016/j.ijpsycho.2012.06.004, PMID: 22705168

[ref54] Xiong G. Liu Y. Liu X. (2014). The meta-analysis on the effect of emotion on rational person behavior in the decision neuroscience perspective. Open Cybern. Syst. J. 8, 1123–1128. 10.2174/1874110X01408011123

[ref55] Yang J. Zhang Q. (2011). Electrophysiological correlates of decision-making in high-risk versus low-risk conditions of a gambling game. Psychophysiology 48, 1456–1461. 10.1111/j.1469-8986.2011.01202.x, PMID: 21913928

[ref56] Yeung N. Botvinick M. Cohen J. D. (2004). The neural basis of error detection: conflict monitoring and the error–related negativity. Psychol. Rev. 111, 931–959. 10.1037/0033-295X.111.4.931, PMID: 15482068

[ref57] Yeung N. Holroyd C. Cohen J. (2005). ERP correlates of feedback and reward processing in the presence and absence of response choice. Cereb. Cortex 15, 535–544. 10.1093/cercor/bhh153, PMID: 15319308

[ref58] Yu R. Luo Y. Ye Z. Zhou X. (2007). Does the FRN in brain potentials reflect motivational/affective consequence of outcome evaluation? Prog. Nat. Sci. 17, 136–143. 10.1080/10020070612331343232

